# Effectiveness of mobile health interventions to improve nasal corticosteroid adherence in allergic rhinitis: A systematic review

**DOI:** 10.1002/clt2.12075

**Published:** 2021-11-16

**Authors:** Mats Stage Baxter, Holly Tibble, Andrew Bush, Aziz Sheikh, Jürgen Schwarze

**Affiliations:** ^1^ Usher Institute The University of Edinburgh Edinburgh UK; ^2^ Asthma UK Centre for Applied Research Usher Institute The University of Edinburgh Edinburgh UK; ^3^ Imperial Centre for Paediatrics and Child Health & National Heart and Lung Institute Imperial College London UK; ^4^ Royal Brompton Hospital London UK; ^5^ Child Life and Health Centre for Inflammation Research The University of Edinburgh Edinburgh UK

**Keywords:** allergic rhinitis, hay fever, medication adherence, mHealth interventions, nasal corticosteroids, Allergische Rhinitis, Heuschnupfen, Medikamentenadhärenz, Mobile Gesundheitsintervention, Nasale Kortikosteroide

## Abstract

**Background:**

Mobile health interventions (MHI) offer the potential to help improve nasal corticosteroid (NCS) adherence in allergic rhinitis (AR). The aim of this systematic review was to summarise the current evidence on the effectiveness of MHI for improving NCS adherence in AR.

**Methods:**

We systematically searched MEDLINE, Embase and the Cochrane Central register of Controlled Trials (CENTRAL) for randomised controlled trials filtered for publication dates between 2010 and 2021. We evaluated the effects of MHI aiming to improve NCS adherence on self‐management outcomes in AR and comorbid conditions. Two reviewers independently screened potential studies, extracted study characteristics and outcomes from eligible papers and assessed risk of bias using the Cochrane Risk of Bias tool 2.0. High heterogeneity precluded meta‐analysis. Data were descriptively and narratively synthesised.

**Results:**

Our searches identified 776 individual studies of which 4 met the inclusion criteria. These studies were heterogeneous with respect to participant, intervention and outcome characteristics. We considered all outcome‐specific overall risk of bias assessments to be of high risk of bias except for two studies examining NCS adherence which received ‘some concern’ grades. The three studies which reported on NCS adherence found that MHI were associated with improvement in NCS adherence. Significant MHI‐associated improvement in symptoms or disease‐specific quality of life was found in one study each, whilst no study reported significant differences in nasal patency.

**Conclusions:**

Whilst MHI showed potential to improve NCS adherence, their effect on clinical outcomes varied. Furthermore, robust studies with longer intervention durations are needed to adequately assess effects of MHI and their individual features on NCS adherence and clinical outcomes.

## 1 BACKGROUND

Allergic rhinitis (AR) is one of the most common diseases globally, estimated to affect over 400 million people; it typically persists throughout life.^1^, ^2^ Because of nasal symptoms (nasal itching, sneezing, rhinorrhoea and nasal congestion), often associated ocular symptoms (itching, tearing and redness of the eye; allergic rhinoconjunctivitis [ARC]) and other related symptoms (itching of the palate, postnasal drip and cough), AR significantly impairs sleep quality and cognitive function, increases discomfort, irritability and fatigue and ultimately reduces disease‐specific quality of life (QoL).^3^ In addition, AR is strongly associated with comorbidities such as asthma^4^, ^5^ and chronic rhinosinusitis (CRS).^6^ As a result, AR causes substantial direct and indirect costs associated with medical expenses and reduction in work and school performance, respectively.^3^

Nasal corticosteroids (NCS) are widely recognised as the most effective medication class for controlling AR symptoms and mitigating their deleterious effects on disease‐specific QoL.^7^, ^8^, ^9^ NCS are the mainstay of AR, ARC and CRS treatment.

NCS usually need to be taken throughout the entire period of allergen exposure to optimally reduce nasal inflammation and AR symptoms^8^, ^10^; however, NCS adherence remains poor and inconsistent for many.^11^

A myriad of underlying factors, including variables related to disease, patient, treatment, physician‐patient relationship and healthcare system contribute to non‐adherence.^12^, ^13^ However, forgetfulness remains one of the principal barriers,^11^, ^14^ suggesting that both intentional and unintentional non‐adherence coexist, in turn necessitating diverse and multifaceted strategies and interventions to effectively improve NCS adherence,^11^ as with other long‐term conditions.^15^

Rapid advances in mobile technologies have ushered mobile health (mHealth) to the fore as a potential tool to improve NCS adherence through the use of a multitude of features that principally promote healthcare professional‐to‐patient and patient‐to‐patient communication, patient empowerment, monitoring and education.^16^ Whilst mHealth represents an intriguing prospect for improving NCS adherence, little clinical research currently exists on its efficacy and benefits.^17^ Moreover, to our knowledge no systematic review has embarked on collating and evaluating current clinical research data.

## 2 OBJECTIVES

To examine whether mHealth interventions (MHI) for improving NCS adherence in AR and comorbid conditions (ARC and CRS) were effective in improving NCS adherence and clinical health outcomes (symptoms and disease‐specific quality of life) compared to usual care not including MHI.

## 3 METHODS

### 3.1 Protocol and registration

The systematic review is registered with, and the corresponding protocol is available from, the PROSPERO database with registration number: CRD42020198879.

### 3.2 Eligible studies

Only randomised controlled trials (RCTs) were eligible for inclusion in the systematic review, including cluster RCTs, wait‐list controlled RCTs and cross‐over RCTs. Quasi‐experimental trials were excluded.

### 3.3 Population

All population groups who were prescribed NCS treatment either as monotherapy or in combination with other treatments for both seasonal and perennial AR with/without ocular symptoms (ARC) or CRS were included. Studies that additionally targeted parents or carers of participants (e.g., children) who contributed to NCS treatment adherence were also included. Individuals exclusively prescribed other treatments excluding NCS (e.g., antihistamines or immunotherapy) were excluded. Interventions which exclusively targeted healthcare professionals were excluded.

### 3.4 Intervention

Studies were included if they delivered interventions with a primary or secondary aim of improving adherence to NCS through the use of MHI. The World Health Organization’s (WHO) definition of mHealth was used for this systematic review, namely a ‘*medical and public health practice* *supported* *by mobile devices, such as mobile phones, patient monitoring devices, personal digital assistants (PDAs) and other wireless devices*’.^18^ Therefore, studies that implemented MHI using mHealth devices, such as mobile phones, smartphones, smartwatches, tablets, PDAs and electronic monitoring devices as an integral part of the intervention were included. Peripheral devices, for example, sensors and sensory wearables and web‐based programmes were included as long as they were accompanied by one or more of the above‐mentioned primary devices. Studies using primary devices that were not handheld or mobile, for example, landline telephones or stationary computers, were not included.

The MHI could be used alone or be part of a broader multifaceted intervention which could be with or without healthcare professional‐to‐patient contact (i.e. face‐to‐face or virtual consultations).

Lastly, studies that exclusively used phone calls or tele‐consultations as an alternative to face‐to‐face consultations were excluded from this systematic review.

### 3.5 Comparators

We only included studies with a control group of participants who were not provided or did not have access to an MHI for improving NCS adherence. Control groups either received usual care or the same intervention devoid of the mHealth component. Usual care pertained to standard care per guidelines or standard care in the given setting at the time in which the study was conducted. Multi‐arm intervention studies, such as varying types of MHI, were included as long as one comparator group matched the criteria above.

### 3.6 Outcomes

The reporting of one or more of the following primary outcomes constituted an inclusion criterion. All relevant study outcomes were extracted upon inclusion.

#### 3.6.1 Primary outcomes:

1. Symptoms as measured by a subjective assessment.
2. Disease‐specific QoL assessed by a validated subjective assessment.
3. Adherence to NCS assessed by objective and/or validated subjective assessments.

#### 3.6.2 Secondary outcomes:

1. Usage of MHI as measured by quantitative usage assessments.
2. Acceptability of MHI using a quantitative instrument, such as questionnaires. Qualitative acceptability assessments were excluded from this systematic review.
3. Nasal patency as measured by an objective test.
4. Adverse effects.

### 3.7 Report eligibility criteria

No restrictions were applied to geographical location or type of setting. Studies written in languages other than English were eligible if they could be translated using Google Translate to a standard where study characteristics were clearly discernible.

Only study reports available in full‐text versions were included. Attempts were made to contact study authors to obtain full‐text articles when unavailable. All supplementary reports or conference abstracts were excluded. Lastly, due to the fast‐paced nature of mHealth research, only studies from 2010 to present were eligible for inclusion.

### 3.8 Information sources

Searches for relevant studies were conducted in MEDLINE (OVID interface), Embase (OVID interface) and CENTRAL (Cochrane Central Register of Controlled Trials; Wiley interface) and were carried out between 28 May 2020 and 27 August 2020 and were refreshed on 15 February 2021. Reference lists of the included studies were scanned in efforts to identify additional relevant publications.

ClinicalTrials.gov, the UK Clinical Research Network Study Portfolio, the Meta Register of Controlled Trials and the first 100 hits on Google Scholar were searched for relevant unpublished or in‐progress trials.

### 3.9 Search strategy

The search strategy was formed using the ‘pearl‐growing’ method in MEDLINE (OVID interface), in which relevant Medical Subject Headings (MeSH), their entry terms and their ‘term‐tree’ were explored, as well as input from the team of authors. The search terms were validated by a medical librarian with expertise in systematic review searching. A draft of the MEDLINE search strategy is presented in Appendix 1 in Supporting Information S1. The MEDLINE search strategy was adapted and translated to the other electronic bibliography databases as to adhere appropriately in syntax and MeSH terms. No search limits or filters were added to individual searches, apart from publishing year range (January 2010–February 2021).

### 3.10 Study selection

Two review authors (MB and HT) were blinded to each other’s verdicts and independently conducted the two‐stage screening of titles and abstracts of study reports extracted from the search results, using the developed screening form (Appendix 2 in Supporting Information S1). Initially, studies that clearly did not meet the inclusion criteria based on their titles were excluded whilst abstracts were scanned against the inclusion criteria during the abstract screening phase. All reports that met the inclusion criteria were coded as ‘Yes’ and otherwise ‘No’ in Covidence,^19^ a systematic review management programme, whilst reasons for exclusion were noted. Where doubt regarding eligibility occurred, these were marked as ‘Maybe’ and were included in the full‐text screening for further scrutiny. Subsequently, all full‐text study reports were retrieved for the studies bearing ‘Yes’ or ‘Maybe’ labels for screening. Additional information was sought from the study authors where necessary to resolve disagreements regarding eligibility or to address uncertainties regarding incomplete or ambiguous methods that required further clarification. Disagreements were resolved either through discussion or by a third review author (JS) whilst reasons for exclusion were documented. Review authors were not blinded to either study authors, journal titles or institutions. Cohen’s kappa for inter‐rater reliability for the title/abstract and full‐text screening were calculated.

### 3.11 Data collection process

A data extraction form (Appendix 3 in Supporting Information S1) was developed and inserted into Covidence. The template for intervention description and replication was used to model the data extraction form.^20^ Two review authors (MB and HT) independently extracted the data from each included study. Both review authors (MB and HT) participated in calibration exercises prior to data extraction. The few disagreements that occurred were resolved through discussion and no arbitrator was needed.

The data extraction form was piloted on one of the included studies and modifications were made where appropriate. Extracted data were divided into the following six distinct domains: general study information, methodology, participant details, intervention details, comparator details and study outcomes.

### 3.12 Data items

The following data items were extracted from the included studies:

1. *General study information*: author(s), institution(s), sponsorship source(s), conflicts of interest, country and setting.
2. *Methods*: study design, date of study, methods of randomisation, length of follow‐up, total study duration, length of ‘run‐in’ period, study centre details, recruitment setting(s) and recruitment methods.
3. *Participants*: number of participants (baseline and follow‐up), gender, median age, range of age, sub‐population groups, condition type(s), condition classification(s), co‐morbidities, inclusion criteria, exclusion criteria, comparison between groups at baseline and mHealth device familiarity.
4. *Interventions*: intervention aim(s) (primary and secondary aims), intervention details, type of intervention(s) (theory or non‐theory‐based), intervention administrator(s), type of mHealth device(s), mHealth device name(s) (e.g., app names), device make(s) and model(s) (if issued), non‐MHI component(s), description of mHealth training (if administered), intervention modification(s), intervention retention and mHealth adherence/usage rates.
5. *Comparison*: comparison group descriptions.
6. *Outcomes*: details about primary and secondary outcomes, including their individual values, data type(s), type of effect measure(s), assessment method(s) and reported time‐points.

Upon completion of data extraction, data was transferred to the review manager RevMan 5^21^ and Microsoft Excel version 16.37 (Microsoft Corporation) by MB whilst being cross‐checked with the study reports by HT.

### 3.13 Risk of bias individual studies

A risk of bias (RoB) assessment for the primary outcomes (NCS adherence, symptoms and disease‐specific QoL) in each study was carried out independently by MB and HT using the Cochrane Risk of Bias tool 2.0^22^ and Microsoft Excel version 16.37 (Microsoft Corporation). We investigated the effect of assignment to intervention (‘intention to treat’). Prior to the assessment, efforts were made to contact study authors to acquire study protocols and trial registry records that were not available to the review authors. The assessment of RoB was conducted using the following domains (as outlined in table 8.2a in the Cochrane Handbook for Systematic Reviews of Interventions)^23^:

1. Bias arising in the randomisation process.
2. Bias due to deviations from intended interventions.
3. Bias due to missing outcome data.
4. Bias in measurement of the outcome.
5. Bias in selection of the reported outcome.
6. Overall bias.

For each domain, a series of ‘signalling questions’ pertaining to the assessment of RoB was answered with either ‘yes’, ‘probably yes’, ‘probably no’, ‘no’ and ‘no information’. An algorithm mapped the recorded answers and proposed a RoB judgement of either ‘low risk of bias’, ‘some concerns’ or ‘high risk of bias’ for each domain. These were overridden by the review authors when deemed appropriate. Comments and direct quotations from study reports were attached to support answers given to each signalling question. Likewise, justification was provided whenever RoB judgements from the algorithm were overridden. Lastly, the domain‐level judgements provided the basis for an overall RoB judgement for each specific outcome being assessed for each study. Review authors were not blinded to study details.

Disagreements were firstly resolved through discussion and secondly via a third review author (AS) for arbitration.

### 3.14 Data synthesis

Study outcome data were not pooled in statistical meta‐analyses due to the clinical heterogeneity of the study characteristics. Instead, the findings were analysed via a narrative synthesis, including tables and figures to aid in data presentation where appropriate.

## 4 RESULTS

### 4.1 Study selection

**FIGURE 1:**
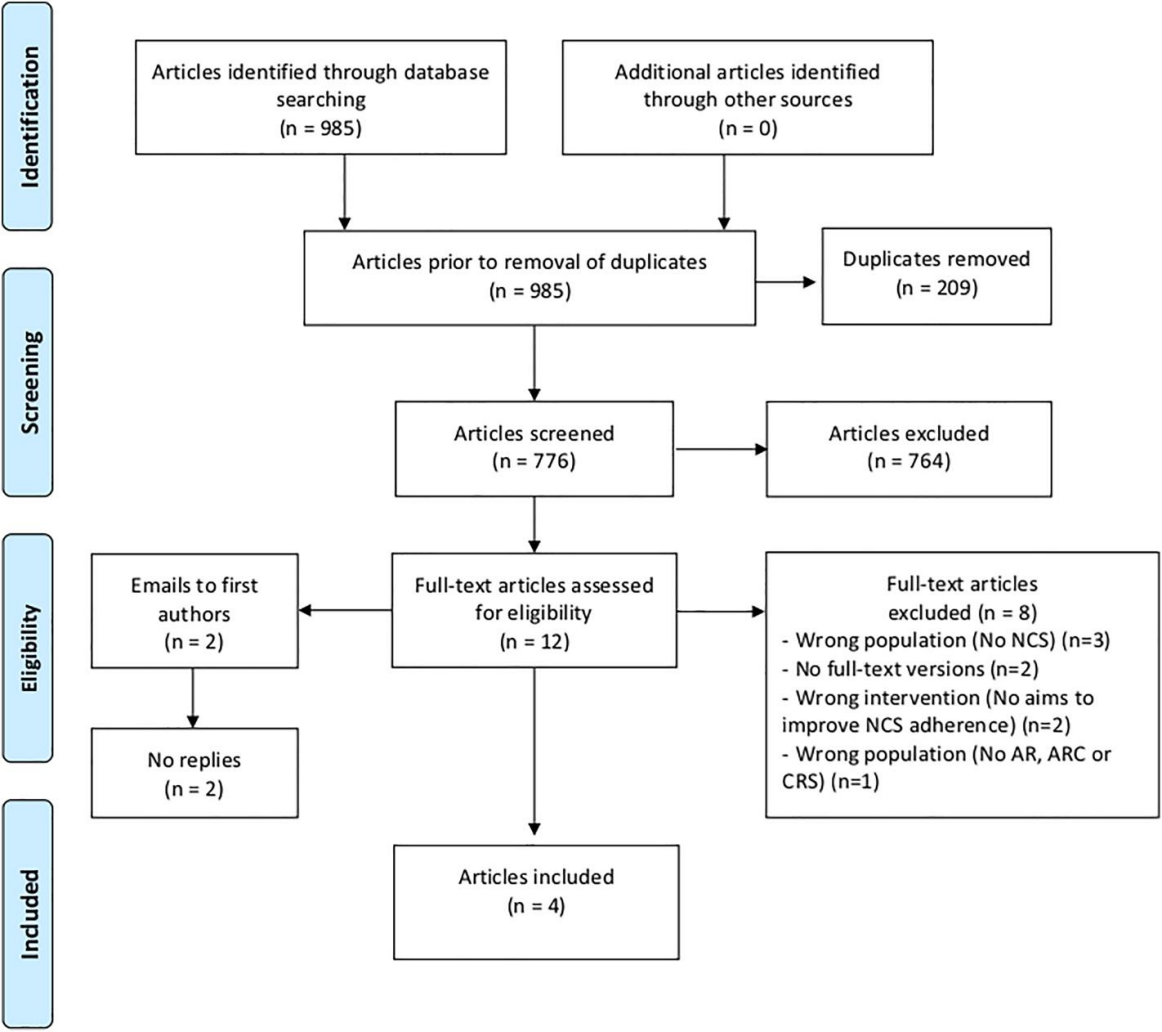
PRISMA flow diagram

The search yielded a total of 985 records as shown in Figure 1. A total of 776 records remained after excluding 209 duplicates.

Subsequently, 764 records which clearly did not adhere to the inclusion criteria were removed during the two‐stage screening process, thereby leaving 12 publications eligible for full‐text review. Two full‐text^24^, ^25^ records could not be procured during the process, despite efforts to contact their respective authors, as these were not available through our institutional holdings. However, upon further examination, it was discovered that both were conference abstracts.

Upon completion of the full‐text review, four studies^26^, ^27^, ^28^, ^29^ were included in the systematic review. The bibliographies were consulted for each of the four included publications, however, no further relevant citations were identified.

The inter‐rater agreement during the title/abstract and full‐text phases produced a Cohen’s kappa of 0.355 (small agreement) and 1.0 (perfect agreement), respectively. The disagreement during the title/abstract mainly stemmed from differing interpretations of the interventions and population groups. However, these were resolved during the subsequent full‐text screening phase.

### 4.2 Characteristics of included studies

#### 4.2.1 Methods

The characteristics of the included studies are summarised in Table 1. The four studies, all two‐arm parallel RCTs, were conducted between 2012 and 2016 in China,^26^, ^29^ Germany^28^ and Turkey^27^ and were all published in English. Three studies^27^, ^28^, ^29^ had intervention durations of 1 month whilst one study^26^ was 3 months.

**TABLE 1:** Characteristics of included studies

Study ID	Study design/condition/setting/country	Participants	Study aims	Interventions	Participants at follow‐up	Outcomes/follow‐up
Cingi et al.^27^	*Study design:* Parallel group RCT *Condition:* AR *Setting:* Outpatient clinic *Country:* Turkey	191 adult patients aged between 21–50 years with persistent AR.	To measure the impact of a mobile patient engagement application on health outcomes in AR, aiming to determine best practices in mobile patient engagement.	I: ‘POPET’ mobile application providing ability to track patient health status, sharing motivational and educational content, whilst also reminding patients to take prescribed NCS (*n* = 95). C: Usual care with limited version of the POPET‐application. Only used for baseline and follow‐up outcome measurements (*n* = 95)	I: 88/96 (91.7%) C: 51/95 (53.7%) Total: 139/191 (72.8%)	*Primary outcome: AR‐specific* QoL *Secondary outcome:* Number of follow‐up visits. *Follow‐up:* 1 month
Feng et al.^26^	*Study design:* Parallel group RCT *Condition:* CRS *Setting:* Tertiary care (hospital) and outpatient clinic *Country:* China	32 adult post functional endoscopic sinus surgery patients with CRS with/without nasal polyps aged 25–56 years.	To improve adherence to NCS in CRS patients following functional endoscopic sinus surgery.	I: WeChat mobile application providing daily information about CRS, importance of intranasal steroid treatment and correct spray technique (*n* = 17) C: Usual care without WeChat application (*n* = 15)	I: 16/17 (94.1%) C: 13/15 (86.7%) Total: 29/32 (90.6%)	*Primary outcome:* NCS adherence *Secondary outcome:* CRS‐specific QoL and endoscopic findings *Follow‐up:* 3 months
Pizzulli et al.^28^	*Study design:* Parallel group RCT *Condition:* ARC *Setting:* Outpatient clinic *Country:* Germany	70 children and adolescents aged between 5–18 years with moderate/severe ARC	To examine whether Internet‐based telemonitoring during the grass‐pollen season of children with allergic rhinoconjunctivitis may enhance adherence to treatment.	I: ‘AllergyMonitor’ (AM) web‐based application with diary function for registering NCS adherence and ARC symptoms, whilst also providing ARC knowledge and pollen count data. SMS alerts were sent as reminders (*n* = 35) C: Usual care without AM application (*n* = 35)	I: 31/35 (88.6%) C: 32/35 (91.4%) Total: 63/70 (90.0%)	*Primary outcome:* NCS adherence *Secondary outcome:* ARC disease control, knowledge of ARC, total nasal flow and total nasal resistance, patient evaluation of the informatics platform. *Follow‐up:* 1 month
Wang et al.^29^	*Study design:* Parallel group RCT *Condition:* AR *Setting:* Outpatient clinic *Country:* China	50 adult AR (persistent/seasonal) patients aged between 18–60.	To evaluate the impact of receiving a daily mobile phone SMS reminder on adherence to treatment and several treatment outcomes in patients with AR.	I: The sending of a daily SMS as a reminder to take prescribed NCS (*n* = 25) C: Usual care without receiving SMS (*n* = 25) All participants received education regarding mechanisms underlying AR symptoms and recommended spray technique	I: 20/25 (80.0%) C: 19/25 (76.0%) Total: 39/50 (78.0%)	*Primary outcome:* NCS adherence *Secondary outcome:* AR symptoms, follow‐up rate, nasal patency and exhaled nNO *Follow‐up*: 1 month

#### 4.2.2 Participants

The total number of participants in the studies was 343, of which 173 and 170 were part of MHI groups and control groups, respectively. All participants were recruited from outpatient care settings; MHI groups ranged between having 17 and 96 participants. Two studies^27^, ^29^ included adults with AR (both perennial/seasonal), one study^26^ included adults with CRS (23/29 had nasal polyps) after functional endoscopic sinusitis surgery, whilst one study^28^ included children and adolescents with ARC (moderate‐to‐severe).

Previous medical histories and skin prick tests were used to determine diagnosis of AR and ARC,^27^, ^28^, ^29^ whilst medical history, nasal endoscopy and CT scan were used to determine CRS.^26^

#### 4.2.3 Interventions

Of the four MHI, two used smartphone apps,^26^, ^27^ one used a web‐based application + Short Message Service (SMS)^28^ and one used SMS^29^ as shown in Table 2. One smartphone app^27^ and web‐based application^28^ used synchronous communication, as two‐way communication was included between participants and physicians, whilst the other smartphone app^26^ and SMS intervention^29^ purely relied on asynchronous communication, exclusively utilising one‐way messaging sent from researchers/physicians to participants.

**TABLE 2:** mHealth intervention features

Study ID	Type of device/communication	NCS log diary/medication reminder	Symptoms log diary	Education	Professional support	Other functions
Cingi et al.^27^	*Delivery:* Smartphone app (POPET) Communication: Synchronous	*NCS log diary:* **✓** Participants could fill in a NCS log diary.	**✓**	**✓**	✓	The RQLQ assessment measured at baseline and follow‐up was a key function on the app itself
		*Medication reminder:* **✓** A daily reminder was sent via the app.	Participants could submit a daily health status on a 7‐point scale (6 = very good and 0 = extremely bad) with an emoticon and share a 140‐character status update	The app both allowed for patient‐to‐care unit correspondence as well as the posting of general information/education messages to the patients.	Participants had a chat function that could send and receive messages with physicians, and ask for immediate assistance with an urgent message option	
Feng et al.^26^	*Delivery:* Smartphone app/social media messenger (WeChat)	*NCS log diary:* **X**	**X**	**✓**	**X**	**X**
	*Communication:* Asynchronous	*Medication reminder:* **✓** A daily reminder was sent via message on WeChat messenger.		Knowledge about CRS, importance of NCS treatment and correct spray technique was delivered via the WeChat social media messenger.		
Pizzulli et al.^28^	*Delivery:* Web‐based app (AllergyMonitor) + SMS	*NCS log diary:* **✓** Participants could fill out a daily electronic diary card on NCS intake.	**✓**	**✓**	**✓**	Daily pollen data was freely available on the web‐based app.
	*Communication:* Synchronous	*Medication reminder:* **✓** A SMS reminder was sent if participants had not inserted medication data for two consecutive days.	Participants could fill out a daily electronic log diary on nasal, ocular and bronchial symptoms	The app delivered knowledge tips about AR and included daily questions on AR knowledge.	Participants could chat with their physician if needed through the web‐based app	
Wang et al.^29^	*Delivery:* SMS	*NCS log diary:* **X**	**X**	**X**	**X**	**X**
	*Communication:* Asynchronous	*Medication reminder:* **✓** A daily reminder was sent via SMS		All participants did receive face‐to‐face information/education on AR, NCS and correct inhaler technique, but was not part of the SMS message itself		

Educational and motivational content, mainly focussing on the importance of adhering to NCS and correct spray technique, was delivered through the mHealth platform in three of four interventions.^26^, ^27^, ^28^ Whilst not delivered through an mHealth device, Wang et al.^29^ delivered face‐to‐face educational content as part of the wider intervention. No studies reported on educational or any other interventional content being based on any behavioural change models. Daily medication adherence reminders were a key function in all the included interventions.

Two interventions included the daily tracking of participants’ symptoms and medication adherence through the mHealth platform.^27^, ^28^

Furthermore, two of the study reports^27^, ^28^ mentioned offering training/walk‐throughs in using the mHealth platforms prior to study commencement.

Lastly, no peripheral devices (e.g., sensory wearables) were used in any of the included studies and all interventions utilised the participants’ own phones throughout the study duration.

#### 4.2.4 Comparisons

In all but one study, the comparators were patients without access to the mHealth platform used in the interventions.^26^, ^28^, ^29^ In the remaining study,^27^ the control group received a limited version of the smartphone app which exclusively enabled participants to complete an electronic AR‐specific quality of life questionnaire (RQLQ) at baseline and follow‐up.

Two^27^, ^29^ studies also reported delivering educational content focused on mechanisms underlying symptoms and recommended use of NCS to the control groups as well.

### 4.3 Primary outcomes

#### 4.3.1 NCS adherence

Three of the four studies reported on NCS adherence^26^, ^28^, ^29^ as shown in Table 3. Of these, one study used a participant‐reported assessment, using number of days being non‐adherent to NCS,^29^ whilst the two other studies utilised objective dose‐count assessments; one based on the amount of spray puffs remaining at follow‐up,^26^ the other on canister weight at follow‐up.^28^

**TABLE 3:** Effects on primary outcomes: nasal corticosteroid adherence, symptoms and quality of life

Outcomes	Reference	Assessment methods	Outcome measures	Outcome summary
NCS adherence	Wang et al.^29^	Self‐reported: The number of days during the study period in which NCS was not taken. Patients were classified as adherent if they reported that they had taken NCS during more than 95% of the study period.	Number of participants >95% adherent: 15/25 (60%) in the intervention group and 7/25 (28%) in the control group were >95% adherent based on the assessment criteria. OR = 3.85 [95% CI: 1.18–12.61] (*p* = 0.02)	The intervention group had a higher proportion of participants classified as being >95% adherent to NCS compared to the control group (15/25 (60%) versus 7/25 (28%)). The participants in the intervention group had nearly fourfold increased odds of being >95% adherent compared to the control group.
	Feng et al.^26^	Dose‐count: Percentage of NCS taken calculated from dose‐count: (120 – rest puffs)/120% × 100%.	Mean adherence rate (%): 93.94% (SE ± 0.71) in the intervention group (*n* = 16), 76.62% (SE ± 0.79) in control group (*n* = 13). Absolute mean difference: 17.30%, (*F* = 118.034, *p* < 0.001).	The intervention group had a higher mean adherence rate compared to the control group (93.94% (SE ± 0.71) versus 76.62% (SE ± 0.79)), producing an absolute mean difference of 17.30% (*F* = 118.034, *p* = < 0.001).
	Pizzulli et al.^28^	Dose‐count: Canister weight at follow‐up	Mean consumption (g/day) of NCS: 0.20 g/day (SD ± 0.12) in the intervention group (*n* = 31) versus 0.15 g/day (SD ± 0.07) in the control group (*n* = 32). Chi‐square (*p* = 0.002), *t*‐test/Mann–Whitney *U* test (*p* = 0.037)	The intervention group had a higher mean intake of NCS (g/day) compared to the control group (0.20 g/day (SD ± 0.12) versus 0.15 g/day (SD ± 0.07)), with strong statistical evidence provided through Chi‐square (*p* = 0.002) and *t*‐test/Mann–Whitney *U* test (0.037) outputs.
Symptoms	Pizzulli et al.^28^	Self‐reported questionnaire: The Allergic Rhinitis Control Test (ARCT)	Mean control test score: 20.1 (SD ± 20.1) mean score in intervention group (*n* = 31) versus 20.8 (SD ± 3.00) mean score in control group (*n* = 32). *t*‐test/Mann–Whitney *U* test was not significant (output not reported)	There was no reported statistical difference in the mean control test score between the intervention group and control group (20.1 (SD ± 20.1) versus 20.8 (SD ± 3.00)). The specific *t*‐test/Mann–Whitney outputs were not significant but specific output were not reported.
	Wang et al.^29^	Self‐reported assessment: Visual Analogue Scales (VAS)	Mean VAS score: 4.38 (SD ± 4.38) in the intervention group (*n* = 25) versus 8.74 (SD ± 6.54) in the control group (*n* = 25). *t*‐test/Mann–Whitney *U* test (*p* = 0.031)	Strong statistical evidence suggested that the intervention group had a significantly lower mean VAS score compared to the control group (4.38 (SD ± 4.38) versus 8.74 (SD ± 6.54)), with a *t*‐test/Mann–Whitney output (*p* = 0.031), favouring the intervention group.
Quality of life	Feng et al.^26^	Self‐reported questionnaire: SinoNasal Outcome Test‐20 (SNOT‐20)	Mean SNOT‐20 Score: 1.50 (SE ± 0.13) in the intervention group (*n* = 16) versus 1.54 (SE ± 0.14) in the control group (*n* = 13). *F*‐test (*F* = 0.043, *p* = 0.988)	There was no reported significant difference in the mean SNOT‐20 score between the intervention group and control group (1.50 (SE ± 0.13) versus 1.54 (SE ± 0.14)), with a *F*‐test (*F* = 0.043, *p* = 0.988)
	Pizzulli et al.^28^	Self‐reported questionnaire: Adolescent/children Rhinoconjunctivitis Quality of Life Questionnaire (AdoIRQLQ)	Mean AdoIRQLQ score: 1.66 (SD 1.66) in intervention group (*n* = 31) versus 1.55 (SD 0.95) in control group (*n* = 32). *t*‐test/Mann–Whitney *U* was not significant (not reported)	There was no reported significant difference in the mean AdoIRQLQ score between the intervention group and control group (1.66 (SD 1.66) versus 1.55 (SD 0.95)). The specific *t*‐test/Mann–Whitney results were not reported on.
	Cingi et al.^27^	Self‐reported questionnaire: Rhinoconjunctivitis Quality of Life Questionnaire (RQLQ)	Median RQLQ score: Median 28.0 (16.0–46.0) in intervention group (*n* = 88) versus Median 39.0 (30.0–55.0) in the control group (*n* = 51). Mann–Whitney *U* test (*p* < 0.001)	The intervention group had a lower median RQLQ score compared to the control group (median 28.0 (16.0–46.0) versus median 39.0 (30–55.0)), with a Mann–Whitney *U* test providing strong evidence to support this difference (*p* < 0.001), favouring the intervention group.

All three trials found strong evidence to suggest that NCS adherence improved among the participants in the intervention groups compared to those of the control groups. More specifically, Feng et al.^26^ found a positive 17.3% absolute mean difference (*F* = 90.88, *p* < 0.001) between mean NCS adherence rates of the intervention and control groups at the end of the study after 3 months. Similarly, Pizzulli et al.^28^ reported strong statistical evidence of association using Chi‐square test and *t*‐test/Mann–Whitney *U* test (χ^2^, *p* = 0.002; *t*‐test/Mann–Whitney *U* test, *p* = 0.037) between NCS adherence and study group, favouring the intervention group.

Wang et al.^29^, using self‐reported days being non‐adherent to NCS, used odds ratio to measure the difference between the intervention and control groups. Implementing an adherence cut‐off at 95%, the intervention group had almost fourfold higher odds of being NCS adherent compared to the control group.

#### 4.3.2 Symptoms

Two of the four studies reported on symptoms, using the five‐item Allergic Rhinitis Control Test (ARCT) questionnaire^28^ and Visual Analogue Scales (VAS)^29^ to assess ARC disease control and AR symptoms, respectively.

Pizzulli et al.^28^ reported no statistical evidence for a difference in the mean ARCT score between trial groups using the Chi‐square test and *t*‐test/Mann–Whitney *U* test (output not reported).

Wang et al.^29^ did find strong evidence to suggest that the intervention group had a significantly improved mean VAS score compared to that of the control group using *t*‐test/Mann–Whitney *U* test (*p* = 0.031).

#### 4.3.3 Quality of life

Three studies reported disease‐specific QoL assessments.^26^, ^27^, ^28^ These included the Rhinoconjunctivitis Quality of Life Questionnaire for adults (RQLQ)^27^ and for adolescents/children (AdolRQLQ)^28^ and the SinoNasal Outcome Test‐20 (SNOT‐20).^26^

Feng et al.^26^ and Pizzulli et al.^28^ did not find any statistical evidence to indicate any difference in disease‐specific QoL scores between intervention and control groups, using the SNOT‐20 (*F*‐test = 0.043, *p* = 0.988) and AdolRQLQ (output not reported) assessments.

Lastly, Cingi et al.^27^ found strong evidence for a positive difference in RQLQ scores between the MHI group and control group using the Mann–Whitney *U* test (*p* < 0.001).

### 4.4 Secondary outcomes

#### 4.4.1 Nasal patency

Two studies reported on nasal patency, measuring nasal airway resistance (NAR) by a rhinomanometer^28^, ^29^ as shown in Table 4. Neither study reported significant differences in NAR between study groups based on Chi‐square test, however, specific statistical outputs were not outlined in the study reports.

**TABLE 4:** Effects on secondary outcomes: Nasal patency, mHealth acceptability and mHealth activity

Outcomes	Reference	Assessment methods	Outcome measures	Outcome summary
Nasal patency	Pizzulli et al.^28^	Objective assessment: Nasal Airway Resistance (NAR) measured by anterior rhinomanometry.	Mean Pa/ml/s: 0.59 (SD ± 0.59) mean NAR in the intervention group (*n* = 31) versus 0.50 (SD ± 0.40) in the control group (*n* = 32). *t*‐test/Mann–Whitney *U* test was not significant (output not reported)	There was no reported statistical difference in mean NAR between the intervention group and control group (0.59 (SD ± 0.59) versus 0.50 (SD ± 0.40)). The specific *t*‐test/Mann–Whitney outputs were not significant but were not reported on.
	Wang et al.^29^	Objective assessment: Unilateral Nasal Airway Resistance (NAR)	Mean Pa/ml/s: 0.17 (SD ± 0.07) mean NAR in the intervention group (*n* = 25) versus 0.19 (SD ± 0.10) in the control group (*n* = 25). *t*‐Test/Mann–Whitney *U* test was not significant (output not reported)	There was no reported statistical difference in mean NAR between the intervention group and control group 0.17 (SD ± 0.07) versus 0.19 (SD ± 0.10)). The specific *t*‐test/Mann–Whitney outputs were not significant but were not reported on.
mHealth acceptability	Cingi et al.^27^	Self‐reported acceptability questionnaire with a 6‐point scale (5 = very good, 0 = extremely bad	Median acceptability score: This assessment was only reported for the intervention group with a median acceptability score of 4.0 (range: 2.0–5.0).	There was no reported acceptability score for the control and therefore no inter‐group comparisons were made.
mHealth activity	Cingi et al.^27^	Platform log registries: Amount of status updates logged into the mHealth platform	Mean status update: This assessment was only reported for the intervention group with a median of status updates of 12 (range: 3.0–27.0).	There were no reported status updates for the control group and therefore no inter‐group comparisons were made.
	Pizzulli et al.^28^	Platform log registries: Number of days actively logging diary data.	Mean percent of days being active: This assessment was only measured for the intervention group with a mean daily diary activity percentage of 74.7% (SD ± 17.5 days, range 40%–100%).	Logging daily diary data was only recorded for the intervention group and therefore no inter‐group comparisons were made.

#### 4.4.2 mHealth acceptability

Only Cingi et al.^27^ reported on participant acceptability of the MHI, which was measured on a 6‐point scale (5 = very good and 0 = extremely bad) for the MHI group only. Out of the maximum six points, the MHI group participants ranked the mHealth device with a median of 4.0 (range: 2.0–5.0).

#### 4.4.3 mHealth activity/usage

Two studies reported on user activity during the trials,^27^, ^28^ however, these were restricted exclusively to the MHI participants. Cingi et al.^27^ reported on the total provision of optional status updates with emoticons during the study duration,^27^ with a median of 12.0 status updates (range, 3.0–89.0). Pizzulli et al.^28^ reported on the mean percent of days logging diary data for symptoms and NCS adherence,^28^ with a mean 74.7% (SD 17.5 days, range 40%–100%) being registered.

#### 4.4.4 Adverse events

No adverse events or harms were reported.

### 4.5 Risk of bias within studies by primary outcomes

**FIGURE 2:**
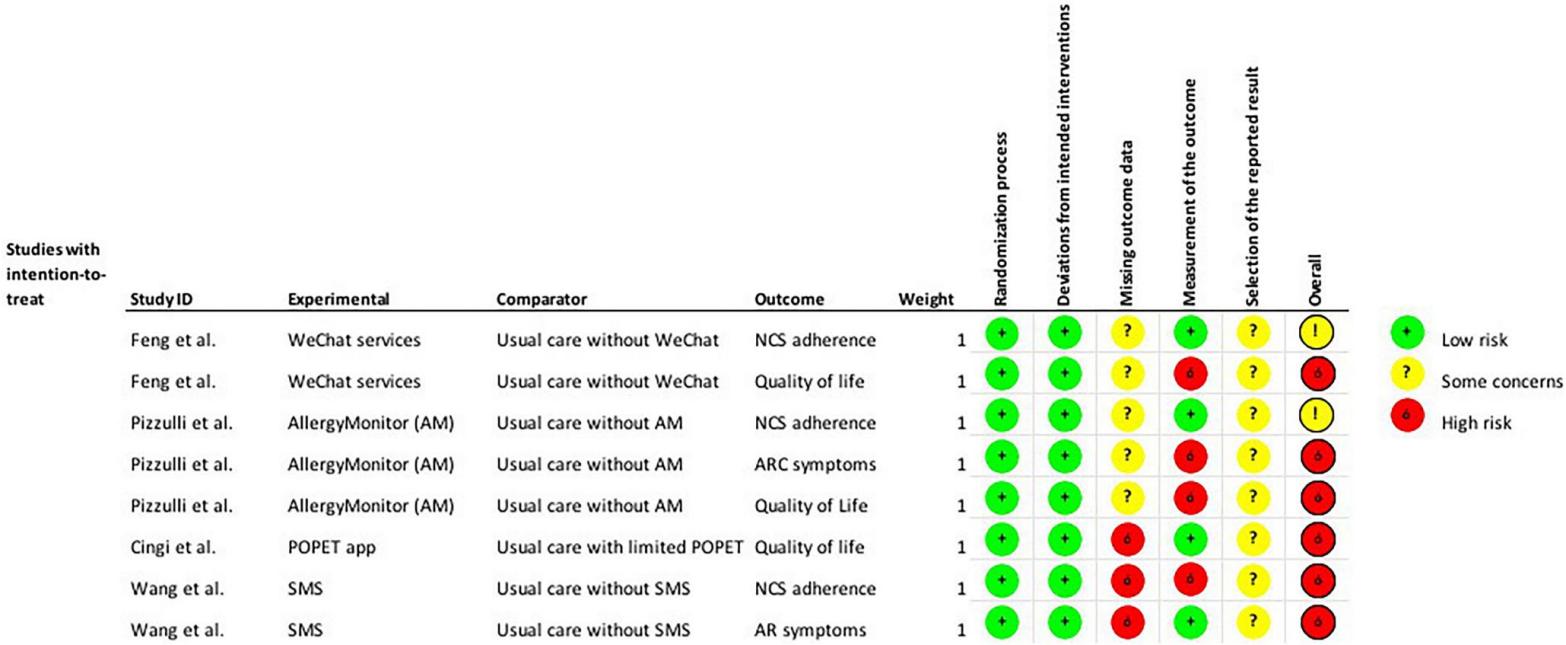
Risk of bias assessment

**FIGURE 3:**
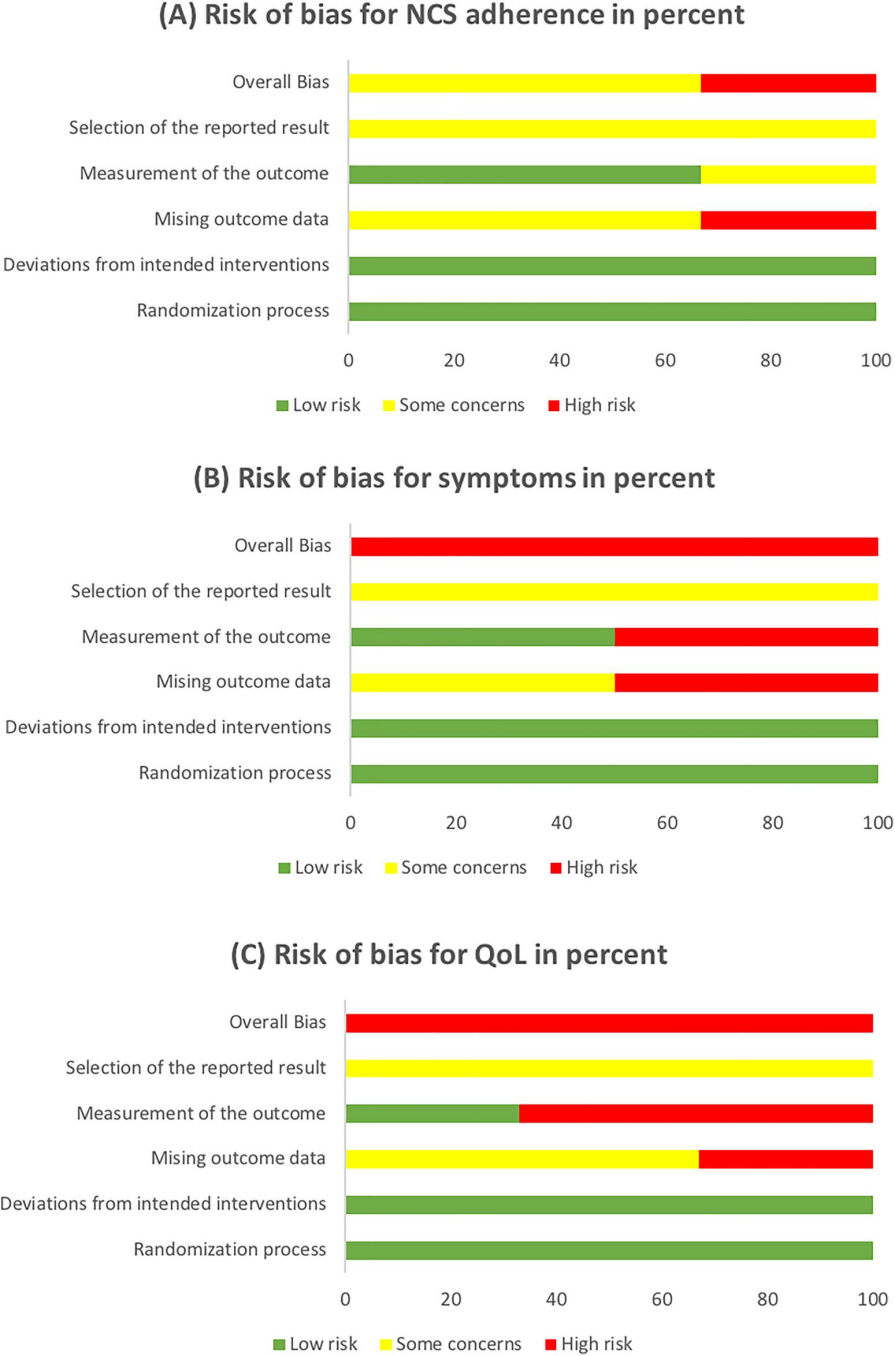
Risk of bias by primary outcome: nasal corticosteroid adherence, symptoms and quality of life

The Cochrane Risk of Bias assessment 2.0^22^ is shown in Figures 2 and 3.

#### 4.5.1 NCS adherence

Among the three trials reporting on NCS adherence, two trials^26^, ^28^ were assessed to have an overall RoB of ‘some concerns’, whilst one^29^ received a ‘high risk of bias’ grade. These were mainly due to exclusion of participants being linked to non‐compliance with treatment,^26^, ^29^ underreporting of reasons for participant exclusion,^28^ inappropriate outcome measurements,^29^ lack of analyses adjusting for missing data^28^ and inavailability of study protocols.^26^, ^28^, ^29^

#### 4.5.2 Symptoms

Concerning symptoms, both trials^28^, ^29^ received an overall ‘high risk of bias’ assessment due mainly to the exclusion of participants being linked to non‐compliance with treatment,^29^ strong likelihood of participants being aware of group allocation when completing participant‐reported assessments,^28^ underreporting of the analyses outputs^28^ and unavailability of study protocols.^28^, ^29^

#### 4.5.3 Disease‐specific QoL

All three trials reporting on disease‐specific QoL received overall ‘high risk of bias’ assessments which mainly were based on significant loss to follow‐up,^27^ lack of analyses adjusting for missing data,^27^ omission of full analyses outputs^28^ and strong likelihood of participants being aware of group allocation when conducting the participant‐reported assessments.^26^, ^28^ Also, all three trials^26^, ^27^, ^28^ did not have available study protocols.

## 5 DISCUSSION

### 5.1 Summary of evidence

This systematic review of MHI for improving NCS adherence in AR, ARC and CRS only identified four RCTs in total.^26^, ^27^, ^28^, ^29^ The findings from the included studies that evaluated NCS adherence suggest that MHI improved NCS adherence across various mHealth platforms (mobile apps, web‐based apps and SMS). Whilst NCS adherence improved between trial groups, less robust findings were reported for symptoms and disease‐specific QoL and no difference was found for nasal patency compared to usual care. No adverse events or harms were reported.

Population groups were diverse as adults with both seasonal and persistent AR, post‐surgery CRS (with or without nasal polyps) and children/adolescents with moderate‐to‐severe ARC were included.

Most of the interventions varied and utilised smartphone apps, web‐based apps and SMS, either exclusively or in combination, whilst mHealth features encompassed medication reminders, medication and symptom diaries, professional support and educational content on nasal spray technique and disease patho‐mechanisms. Whilst primary outcome variables did overlap, as either medication adherence, symptoms and disease‐specific QoL were assessed in every study, their assessment methods differed considerably.

mHealth acceptability ratings were recorded as good, albeit in a single study, whilst differing measures of mHealth activity generally were reported to be high in two studies.

One notable additional outcome, not pre‐specified in the review protocol, was ARC knowledge, assessed by questionnaire by Pizzulli et al.^28^ which improved in the MHI group with a higher rate of correct answers compared to usual care (83.3% vs. 67.7%, *p* < 0.001).

### 5.2 Strengths and limitations

Our systematic review had a number of strengths and limitations. The strengths included the use of an expansive search strategy which yielded 776 unique records. Two reviewers independently screened, extracted data and assessed the quality of studies, with the latter being carried out with a novel version of a validated and compendious RoB tool.

However, this study also had some limitations.

### 5.3 Outcome level limitations

Apart from two ‘some concerns’ overall RoB assessments for NCS adherence, all reported outcome results were deemed to be of high risk of bias. The main issues included a lack of participant blinding in participant‐reported outcome assessments, participant exclusions possibly being linked to interventions, significant loss to follow‐up, lack of intention‐to‐treat analyses, incomplete outcome data and unavailability of study protocols. Moreover, the lack of objective NCS adherence measures that reliably track participants’ adherence patterns between study start and follow‐up visits should be viewed as a significant limitation.

In general, there was a lack of power due to small participant numbers and short study durations which might especially have had an influence on the varied results for disease‐specific QoL, symptoms and nasal patency as longer time periods may be required to see the full effects of sustained uptake of NCS.^30^

The clinical importance of the findings was also difficult to ascertain as no information on clinically relevant differences or effect sizes were provided.

Moreover, due to the considerable heterogeneity across study characteristics and reported outcome measures, it was not possible to perform meta‐analyses.

The small number of included studies meant that sensitivity analyses were not feasible, limiting the evidence of the effects of individual mHealth features, mHealth activity and acceptability on adherence and clinical outcomes.

In a constantly evolving mHealth field, no study was conducted beyond 2016, therefore the effects of more contemporary MHI are also less clear.

### 5.4 Review level limitations

Only three bibliography databases were used for this review, potentially leaving relevant studies undetected. Also, not using country/region‐specific databases might have introduced language bias. However, MEDLINE, Embase and CENTRAL are considered the most integral databases to search for reports of trials by the Cochrane review group.^23^

Despite contacting the authors, we were unable to retrieve two full‐text articles^24^, ^25^ during the full‐text screening phase, which could be a limitation. However, these were conference abstracts and are not likely to be available in full‐text versions.

### 5.5 Interpretation of results

To our knowledge, no systematic review on MHI effectiveness has been conducted in AR, ARC or CRS. However, our findings are in line with a systematic review by Miller et al.^31^ who reported MHI (mobile apps and SMS) were efficacious in improving inhaled corticosteroid (ICS) adherence in asthma compared to usual care, whilst mixed results were found for disease‐specific QoL and asthma symptoms. Although the meta‐analysis did provide a positive cumulative standardised mean difference for disease‐specific QoL, one of three studies reported no improvement. Likewise, a systematic review^32^ examining the effectiveness of reminder systems (web‐based apps and SMS) for ICS adherence in asthma, found similar improvements in ICS adherence whilst no differences were reported for disease‐specific QoL and asthma symptoms compared to usual care. As with the current review, both reviews reported low numbers of included studies and short study durations as significant limitations.

As both unintentional and intentional non‐adherence exist in both AR and asthma, a multitude of strategies may be needed to effectively improve adherence.^11^ In particular, education is seen as a key tool in addressing intentional non‐adherence in AR and asthma,^11^, ^33^ especially if underpinned by behavioural change models and supported by technology.^34^ While educational components were present in all but one of the included interventions, however, the use of behavioural change models were not reported in any of the included studies.

Overall, incorporating a diverse range of interventional components, as included in the current review, including education, self‐monitoring, reinforcement, professional support and reminders could improve NCS adherence in both AR and comorbid conditions.

### 5.6 Implications and recommendations

Our findings indicate that MHI have the potential to improve NCS adherence in AR, ARC and CRS across various mHealth platforms, whilst their subsequent effects on clinical outcomes remain less clear compared to usual care. However, the current evidence base is weak and somewhat outdated in the fast‐paced field of mHealth. Larger, more robust studies with longer durations and information pertaining to the clinical importance of findings are needed to improve the applicability to patients and healthcare providers alike.

As multifaceted interventions may help target the complex multifactorial nature of non‐adherence to NCS, future research should carefully evaluate the efficacy of individual MHI components to help better determine the most effective combinations of mHealth features. In addition, the efficacy of behavioural change models in designing educational components should be further elucidated, including measuring the effect on disease‐specific knowledge.

## 6 CONCLUSIONS

The current review highlights both the potential effectiveness of MHI for improving NCS adherence in AR, ARC and CRS and a range of methodological issues within the current evidence base and thus a need for future research to fill important evidence gaps. Due to the relative infancy of the field and current research dearth, more robust studies are needed to properly evaluate the long‐term efficacy of MHI and their sub‐components on NCS adherence and clinical outcomes in AR, ARC and CRS. It will also be important to understand if MHI for these conditions also affect outcomes of comorbid asthma.

## CONFLICT OF INTERESTS

Mats Stage Baxter received funding from the Chief Scientist Office. Jürgen Schwarze received speaker honorarium from Mylan, consulting fees from Aimune. Holly Tibble declares that she has no conflicts of interest. Andrew Bush declares that he has no conflicts of interest. Aziz Sheikh received grants from Asthma UK.

## Supporting information

Supporting Information S1
