# Supplementary material for: Effectiveness of mobile health interventions to improve nasal corticosteroid adherence in allergic rhinitis: A systematic review
Source: Clin Transl Allergy. 2021 Nov 16;11(9):e12075. doi: 10.1002/clt2.12075 (PMC9815425; doi:10.1002/clt2.12075)
Supplement: Supplementary file 1 — Supporting Information S1 [file CLT2-11-e12075-s001.docx]

**Appendix**

**Appendix 1.** Search strategy in MEDLINE

| #1 | MESH Allergic Rhinitis EXPLODE ALL |
| --- | --- |
| #2 | MESH Allergens EXPLODE ALL |
| #3 | MESH Allergic Conjunctivitis EXPLODE ALL |
| #4 | MESH Sinusitis EXPLODE ALL |
| #5 | MESH Nose Diseases EXPLODE ALL |
| #6 | (“hay fever” OR hayfever OR allergy OR allergies OR allergic OR rhinitis OR “allergic asthma” OR rhinoconjunctivitis OR rhinosinusitis OR rhinorrhea OR rhinopathy OR “upper airway disease*”):ti,ab,kw. |
| #7 | #1 OR #2 OR #3 OR #4 OR #5 OR #6 |
| #8 | MESH Telemedicine EXPLODE ALL |
| #9 | MESH Smartphone EXPLODE ALL |
| #10 | MESH Mobile Applications EXPLODE ALL |
| #11 | MESH Wearable Electronic Devices EXPLODE ALL |
| #12 | MESH Cell Phone EXPLODE ALL |
| #13 | MESH Text Messaging EXPLODE ALL |
| #14 | MESH Social Media EXPLODE ALL |
| #15 | MESH Reminder Systems EXPLODE ALL |
| #16 | MESH Internet-based Intervention EXPLODE ALL |
| #17 | MESH Remote Sensing Technology EXPLODE ALL |
| #18 | MESH Minicomputers EXPLODE ALL |
| #19 | MESH Microcomputers EXPLODE ALL |
| #20 | MESH Video Games EXPLODE ALL |
| #21 | (tele-med* OR tele-health* OR telehealth* OR mHealth OR m-health OR “m health” OR “mobile health” OR mobile-health OR “mobile technolog*” OR “mobile solution” OR smartphone* OR “mobile intervention” OR mobile device OR mobile instrument* OR wearables OR mobile sensor* OR cell phone OR telemonitoring OR tele-monitoring OR SMS OR “text message*” OR “short message*” OR texting OR MMS OR wireless OR Bluetooth):ti,ab,kw. |
| #22 | (ehealth OR e-Health OR digital health* OR web-based):ti,ab,kw. |
| #23 | (Facebook OR Twitter OR Instagram OR Snapchat OR WhatsApp OR WeChat):ti,ab,kw. |
| #24 | #8 OR #9 OR #10 OR #11 OR #12 OR #13 OR #14 OR #15 OR #16 OR #17 OR #18 OR #19 OR #20 OR #21 OR #22 OR #23 |
| #25 | MESH Medication Adherence EXPLODE ALL |
| #26 | MESH Self Medication EXPLODE ALL |
| #27 | MESH Self-Management EXPLODE ALL |
| #28 | (adherence OR compliance OR corticosteroid* OR ((treatment OR medication) AND (regime OR plan OR intake)) OR (controller AND (medication OR treatment))):ti,ab,kw. |
| #29 | (((intranasal OR nasal) AND (steroid* OR corticosteroid*)) OR corticosteroid* OR NCS OR aerosol OR spray OR Mometasone OR Fluticasone OR Beclomethasone OR Budesonide OR Ciclesonide OR Triamcinolone OR medication OR medications OR prescribed):ti,ab,kw. |
| #30 | #25 OR #26 OR #27 OR #28 OR #29 |
| #31 | #7 AND #24 AND #30 |

**Appendix 2.** Screening form

| Study elements | Are inclusion criteria met? | | Reasons for exclusion |
| --- | --- | --- | --- |
| *Study design:* Is the study design an RCT? | Yes /  unclear | No | 1. The study design is not an RCT. |
| *Population:* Does the study enrol participants that are prescribed nasal corticosteroids for AR, ARC or CRS? | Yes /  unclear | No | 1. Study does not enrol participants on prescribed nasal corticosteroids for AR, ARC or CRS. |
| *Intervention:*   - Are appropriate mHealth devices used as an integral part of the intervention? - Does the study intervention have primary or secondary aims at improving nasal corticosteroid adherence? | Yes /  unclear | No | 1. Appropriate mHealth devices are not used as an integral part of the intervention. 2. The study intervention does not have primary or secondary aims at improving nasal corticosteroid adherence. |
| *Outcomes: Do the outcomes of the study include one or more of the following:*   - Symptoms as measured by a subjective assessment. - Quality of life (QoL) assessed by a validated subjective assessment. - Adherence to nasal corticosteroids assessed by objective or validated subjective assessments. | Yes /  unclear | No | 1. No study outcomes included one or more of the following:  - Symptoms as measured by a subjective assessment. - Quality of life (QoL) assessed by a validated subjective assessment. - Adherence to nasal corticosteroids assessed by objective or validated subjective assessments. |

**Appendix 3.** Data extraction form

| **General Study Information** | |
| --- | --- |
| **Study Citation** |  |
| **Author(s)** |  |
| **Institution(s)** |  |
| **Sponsorship source(s)** |  |
| **Conflicts of interest** |  |
| **Country** |  |
| **Study setting** |  |
| **Methods** | |
| **Study design** |  |
| **Study date** |  |
| **Methods for random sequence generation** |  |
| **Details on allocation sequence concealment** |  |
| **Details on blinding** |  |
| **Length of follow-up** |  |
| **Total study duration** |  |
| **”run-in” period length (if any)** |  |
| **Amount of study centres** |  |
| **Study centre details** |  |
| **Recruitment setting (if different from study setting)** |  |
| **Recruitment method(s)** |  |
| **Participants** | |
| **Sample size (N) at baseline** |  |
| **Sample size (N) at completion** |  |
| **Number/% female/male** |  |
| **Median age** |  |
| **Age range** |  |
| **Sub-population group** |  |
| **Severity of AR/ARC or CRS** |  |
| **Number/% with other respiratory diseases** |  |
| **Inclusion criteria** |  |
| **Exclusion criteria** |  |
| **Comparison between groups at baseline** |  |
| **Number/% Familiar with mHealth devices** |  |
| **Intervention(s)** | |
| **Intervention aim(s) (primary and secondary)** |  |
| **Intervention details** |  |
| **Type of intervention (theory or non-theory-based)** |  |
| **Intervention administrators** |  |
| **Type of mHealth devices** |  |
| **mHealth device name(s)** |  |
| **Phone/Device make and model (if issued)** |  |
| **Non-mHealth components** |  |
| **Description of mHealth training (if administered)** |  |
| **Intervention retention (N/%)** |  |
| **mHealth device adherence/usage rates (in Numbers/% or classification)** |  |
| **Intervention modifications** |  |
| **Comparator(s)** | |
| **Comparison group(s) description** |  |
| **Outcomes (for each outcome measure)** | |
| **Values for each outcome (Baseline)** |  |
| **Values for each outcome (T1)** |  |
| **Values for each outcome (T2)** |  |
| **Values for each outcome (T3)** |  |
| **Data type (dichotomous/continuous)** |  |
| **Type of effect measure (e.g., OR, mean difference)** |  |
| **Assessment method** |  |
| **Reported time-points** |  |
